# Amikacin Optimal Exposure Targets in the Hollow-Fiber System Model of Tuberculosis

**DOI:** 10.1128/AAC.00961-16

**Published:** 2016-09-23

**Authors:** Shashikant Srivastava, Chawanga Modongo, Chandima W. Siyambalapitiyage Dona, Jotam G. Pasipanodya, Devyani Deshpande, Tawanda Gumbo

**Affiliations:** a Center for Infectious Diseases Research and Experimental Therapeutics, Baylor Research Institute, Baylor University Medical Center, Dallas, Texas, USA; b Division of Infectious Diseases, University of Pennsylvania, Philadelphia, Pennsylvania, USA; c Botswana-University of Pennsylvania Partnership, Gaborone, Botswana; d Department of Medicine, University of Cape Town, Observatory, Cape Town, South Africa

## Abstract

Aminoglycosides such as amikacin are currently used for the treatment of multidrug-resistant tuberculosis (MDR-TB). However, formal pharmacokinetic/pharmacodynamic (PK/PD) studies to identify amikacin exposures and dosing schedules that optimize Mycobacterium tuberculosis killing have not been performed. It is believed that aminoglycosides do not work well under acidic conditions, which, if true, would mean poor sterilizing activity against semidormant bacilli at low pH. We performed time-kill studies to compare the bactericidal effect of amikacin in log-phase-growth bacilli with the sterilizing effect in semidormant bacilli at pH 5.8 in broth. In log-phase M. tuberculosis at normal pH versus semidormant M. tuberculosis at pH 5.8, the maximal kill (*E*_max_) estimate and 95% confidence interval (CI) were 5.39 (95% CI, 4.91 to 5.63) versus 4.88 (CI, 4.46 to 5.22) log_10_ CFU/ml, while the concentration mediating 50% of *E*_max_ (EC_50_) was 1.0 (CI, 0. 0.86 to 1.12) versus 0.60 (CI, 0.50 to 0.66) times the MIC, respectively. Thus, the optimal exposures and kill rates identified for log-phase M. tuberculosis will be optimal even for semidormant bacilli. Next, we performed exposure-response and dose-scheduling studies in the hollow-fiber system model of tuberculosis using log-phase M. tuberculosis. We recapitulated the amikacin concentration-time profiles observed in lungs of patients treated over 28 days. The PK/PD index linked to M. tuberculosis kill was the peak concentration (*C*_max_)-to-MIC ratio (*r*^2^ > 0.99), closely followed by the area under the concentration-time curve from 0 to 24 h (AUC_0–24_)-to-MIC ratio (*r*^2^ = 0.98). The EC_90_ was a *C*_max_/MIC ratio of 10.13 (95% CI, 7.73 to 12.48). The EC_90_ is the dosing target for intermittent therapy that optimizes cure in TB programs for MDR-TB patients.

## INTRODUCTION

Multidrug resistant tuberculosis (MDR-TB) is difficult to treat and has poor outcomes (1). Several agents were recently licensed or repurposed to treat MDR-TB, including bedaquiline, delamainid, and oxazolidinones (2–4). However, aminoglycosides and quinolones still constitute the backbone of “optimized background regimens” (5). Until now, the aminoglycoside exposures and dose schedules associated with maximal efficacy in the treatment of MDR-TB have been unexplored. In aminoglycoside treatment of Gram-negative bacilli, the peak concentration (*C*_max_)-to MIC ratio were found to be linked to efficacy, which led to a revolutionary overhaul of aminoglycoside regimens and dramatic improvement in clinical outcomes (6, 7). Here, we used a preclinical hollow-fiber system model of tuberculosis (HFS-TB) to identify dose-effect and dose-scheduling relationships of amikacin against Mycobacterium tuberculosis (8, 9). The HFS-TB has a forecasting accuracy of within 94% of the drug exposures later identified in TB patients in clinical studies after the fact, and it has been qualified as a drug development tool by the European Medicines Agency and endorsed by the Food and Drug Administration (9–13). Thus, the identified amikacin exposures associated with maximal kill are expected to be clinically relevant to MDR-TB patients.

## MATERIALS AND METHODS

### Bacterial strains, materials, and reagents.

M. tuberculosis H37Ra (ATCC 25177) was procured from American Type Culture Collection (ATCC), Manassas, VA. Prior to each experiment M. tuberculosis stock was thawed and grown in Middlebrook 7H9 broth supplemented with 10% oleic acid-dextrose-catalase (OADC) (here termed “broth”) at 37°C under 5% CO_2_ and shaking conditions for 4 days to achieve log-phase growth (LPG). Methicillin-resistant Staphylococcus aureus (MRSA) (ATCC 33591) was purchased from ATCC. Prior to each experiment, MRSA was grown from stock, and a single colony was picked from the Mueller-Hinton agar plate and grown in Mueller-Hinton broth with rapid shaking at 37°C for 3 h. This log-phase MRSA culture was used in subsequent experiments.

Hollow-fiber cartridges were purchased from FiberCell (Frederick, MD). Amikacin was purchased from the Baylor University Medical Center pharmacy. Streptomycin and pyrazinamide were purchased from Sigma-Aldrich (USA). The drugs were serially diluted using broth to the drug concentrations required for study.

### Identification of M. tuberculosis amikacin MIC.

M. tuberculosis MICs were identified using standard broth methods (14). The amikacin MIC was also independently identified using an Etest strip on Middlebrook 7H10 agar supplemented with 10% OADC (here termed “agar”). Tests were performed twice. The amikacin mutation frequency was determined by culturing 0.2 ml of 10^8^ CFU/ml M. tuberculosis on 20 agar plates supplemented with 6× MIC.

### Amikacin concentration effect studies at normal and acidic pH in test tubes.

M. tuberculosis cultures on day 4 of LPG at normal broth pH or semidormant M. tuberculosis cultures in broth acidified to pH 5.8 using citric acid (SDB) were adjusted to a final bacterial density of 1.5 × 10^5^ CFU/ml and 9 ml of broth dispensed to test tubes (15). The pH of 5.8 was chosen based on our extensive experiments in the past (15). In all experiments, the final pH was verified using an Oakton pH 150 meter (Oakton Instruments, Vernon Hills, IL, USA). One milliliter of amikacin solution in broth was then added to make final concentrations of 0, 0.125, 0.25, 0.5, 1, 2, 4, and 8 mg/liter, in triplicate, after which the cultures were incubated at 37°C under 5% CO_2_ with slow shaking. On day 7, the cultures were washed twice to remove drug carryover, serially diluted, and cultured on agar, after which colonies were counted. In order to make sure that an effect at low pH was not an artifact of Middlebrook broth or citric acid, we repeated the exposure effect experiments using Sauton's medium. We acidified the medium to a pH of 5.8 by titrating hydrochloric acid. In addition, we also used 100 mg/liter of pyrazinamide (which is known to kill M. tuberculosis at acidic pH) as a positive control for effect at low pH. The inhibitory sigmoid *E*_max_ model, where *E*_max_ is maximal kill, was employed to identify the relationship between bacterial CFU per ml and drug concentration.

Since the notion that aminoglycosides do not kill under acidic conditions arose during streptomycin's halcyon days, we also explored that drug's activity at low pH in order to see if the pH effects were the same for the entire pharmacophore. Studies were performed using MRSA for a more rapid readout. LPG MRSA was coincubated with amikacin and streptomycin at final concentrations of 0, 0.03, 0.06, 0.125, 0.25, 0.5, 1, and 2 mg/liter in Mueller-Hinton broth for 4 h with rapid shaking at 35°C. The cultures were incubated in Mueller-Hinton broth either at neutral pH (7.3) or at pH 5.0 after acidification with citric acid. After 4 h, the cultures were washed twice with normal saline to remove the carryover drug, serially diluted, and incubated overnight on Mueller-Hinton agar to enumerate the total bacterial burden. The experiment was performed twice with three replicates for each drug concentration.

### Dose-response and dose-scheduling hollow-fiber studies.

The HFS-TB has been described in detail elsewhere, with steps and standards for PK/PD studies enumerated previously (8, 9). M. tuberculosis was grown into LPG and 20 ml (10^6^ CFU/ml) inoculated into the peripheral compartments of 16 HFS-TB systems. The systems were treated with amikacin concentration-time profiles that mimicked those achieved in the lungs of TB patients (16, 17). Amikacin was administered over 30 min from computer-controlled syringe pumps to the central compartment of each HFS-TB in order to recapitulate the drug's time to maximum concentration in patients. Eight exposures, covering drug concentration-time profiles achieved by human doses of 0, 0.35, 0.75, 1.25, 2.5, 5, 10, 20, and 30 m/kg/day, were administered, assuming negligible protein binding given amikacin's protein binding of <5%. In order to break the collinearity of the *C*_max_/MIC ratio, the area under the concentration-time curve from 0 to 24 h (AUC_0–24_)-to-MIC ratio, and the percentage of time that the concentration persists above the MIC (%*T*_MIC_), we used the results of our test tube experiments to administer six of these drug exposures (human equivalents of 1.25, 2.5, 5, 10, 20, and 30 mg/kg) to 6 extra HFS-TB systems at a dosing schedule of once every 48 h (in addition to the once-a-day schedule) and the 30 m/kg on a twice-a-day dosing schedule. These exposures were chosen so that in addition to the dose fractionation exercise, we could also explore the exposure effect over a large enough dynamic range of either *C*_max_/MIC ratio, AUC_0–24_/MIC ratio, %*T*_MIC_, or trough (*C*_min_)/MIC ratio. Since we use the actual exposures achieved in the HFS-TB for PK/PD analyses, we sampled the central compartment of each HFS-TB at 0.5, 2.5, 5, 7.5, 10, 15, and 23.5 h after the first dose. Assays for amikacin concentration identification were as described in the past (18). In order to quantitate the M. tuberculosis burden, we sampled the peripheral compartment of each HFS-TB on days 3, 7, 14, 21, and 28. Samples were washed and processed as described above for culture on agar. In addition, in order to enumerate the amikacin-resistant subpopulation, the cultures were also incubated on agar supplemented with 6 times the amikacin MIC. The cultures were incubated for 4 weeks.

### PK and PD modeling.

Compartmental pharmacokinetic modeling was performed using steps outlined in our prior publications (19, 20). The pharmacokinetic parameter estimates were used to calculate the observed amikacin exposures of *C*_max_/MIC, AUC_0–24_, AUC_0–24_/MIC, %*T*_MIC_, and *C*_min_/MIC exposures. Microbial effect versus amikacin exposure was examined using the inhibitory sigmoid *E*_max_ model. The PK/PD index that best described microbial kill was chosen using Akaike information criteria (AIC) (21). We calculated likelihood ratios for choosing one PK/PD index over another using the difference in AIC scores (21). From the inhibitory sigmoid *E*_max_ relationship we calculated the concentration mediating 90% of *E*_max_ (EC_90_), which is the exposure mediating optimal effect (9–11, 22). All analyses were performed using ADAPT 5 software (23).

## RESULTS

**FIG 1:**
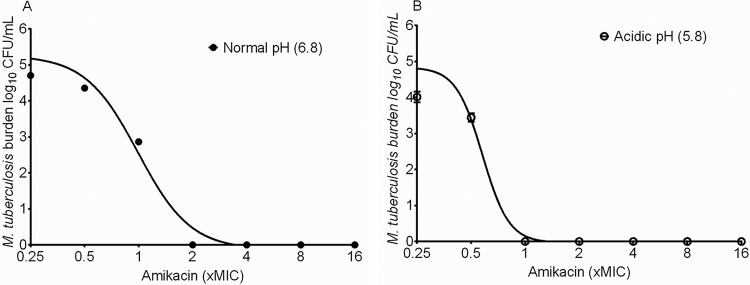
Amikacin efficacy against M. tuberculosis at different pHs. The maximal kill of log-phase-growth M. tuberculosis by amikacin at normal pH (A) was similar to that in slowly growing M. tuberculosis at low pH (B). The figure shows that maximal kill was actually achieved at lower concentrations at low pH.

The amikacin MIC against M. tuberculosis was 0.5 mg/liter on two separate occasions, using two separate methods. The amikacin mutation frequency was 2 × 10^−7^. The inhibitory sigmoid *E*_max_ relationship between amikacin and LPG M. tuberculosis is shown in Fig. 1A. At the Middlebrook broth pH of 6.8 as measured during the experiment, the amikacin *E*_max_ was 5.39 (95% confidence interval [CI], 4.91 to 5.63) log_10_ CFU/ml, the Hill slope (*H*) was 2.96 (95% CI, 1.89 to 4.04), and the EC_50_ was 0.99 (95% CI, 0.86 to 1.12) times the MIC (*r*^2^ = 0.98). Figure 1B shows that against SDB at pH 5.8, the *E*_max_ was 4.87 (95% CI, 4.39 to 5.35) log_10_ CFU/ml, *H* was 5.88 (95% CI, 1.19 to 10.57), and the EC_50_ was 0.58 (95% CI, 0.49 to 0.66) times the MIC (*r*^2^ = 0.97). Thus, *H* and *E*_max_ were the same at either pH since the confidence intervals overlapped; however, the EC_50_ was actually better at the lower pH. In order to make sure that the amikacin effect at acidic pH was not peculiar to Middlebrook medium and citric acid, we performed the same concentration effect experiment in Sauton's medium acidified to pH 5.8 using HCl. The nontreated controls grew from 5.70 ± 0.04 log_10_ CFU/ml to 7.10 ± 0.14 log_10_ CFU/ml in 7 days. The positive control, 100 mg/liter pyrazinamide, killed 0.73 log_10_ CFU/ml of M. tuberculosis in the first 7 days compared to nontreated controls. The lowest amikacin concentration, 0.03 mg/liter, was able to kill 5.70 log_10_ CFU/ml M. tuberculosis in 7 days at pH 5.8 in Sauton's medium.

**FIG 2:**
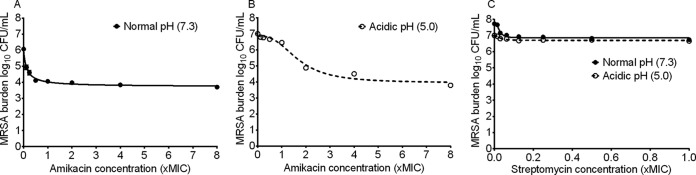
Effect of pH on aminoglycoside efficacy against MRSA. (A) Amikacin had a good *E*_max_ against MRSA at normal pH. (B) The amikacin *E*_max_ was higher at pH 5.0. In other words, amikacin efficacy was not dependent upon the pH of the growth medium. (C) However, the streptomycin *E*_max_ and EC_50_ were altered by the pH changes.

The amikacin MIC against MRSA was 0.25 mg/liter. Concentration effect experiments with MRSA revealed the results shown in Fig. 2. At a documented pH of 7.3 in Mueller-Hinton broth, the *E*_max_ was 2.34 (95% CI, 2.15 to 2.52) log_10_ CFU/ml, *H* was 0.94 (95% CI, 0.66 to 1.22), and the EC_50_ was 0.12 (95% CI, 0.09 to 0.15) times the MIC (*r*^2^ = 0.98) (Fig. 2A). Figure 2B shows that at a pH of 5.0, the *E*_max_ was 2.92 (95% CI, 2.61 to 3.22) log_10_ CFU/ml, *H* was 2.70 (95% CI, 0.81 to 3.59), and the EC_50_ 1.68 (95% CI, 1.44 to 1.92) times the MIC (*r*^2^ = 0.97). Thus, while *E*_max_ does not change, the EC_50_ against MRSA was significantly poorer under acidic conditions, which differs from M. tuberculosis exposure effect findings. Figure 2C compares the exposure effect findings for streptomycin against MRSA (streptomycin MIC = 2 mg/liter) at two different pHs in Mueller-Hinton broth. At pH 7.3 the *E*_max_ was 0.89 (95% CI, 0.76 to 1.03) log_10_ CFU/ml, *H* was 2.79 (95% CI, 1.41 to 4.18), and the EC_50_ was 0.03 (95% CI, 0.02 to 0.03) times the MIC (*r*^2^ = 0.94). However, consistent with the long-standing belief, at pH 5.0, the streptomycin *E*_max_ fell to 0.32 (95% CI, 0.25 to 0.38) log_10_ CFU/ml, but *H* was 1.89 (5% CI, 0.79 to 2.96) and the EC_50_ was still 0.03 (95% CI, 0.02 to 0.04) times the MIC (*r*^2^ = 0.89).

**FIG 3:**
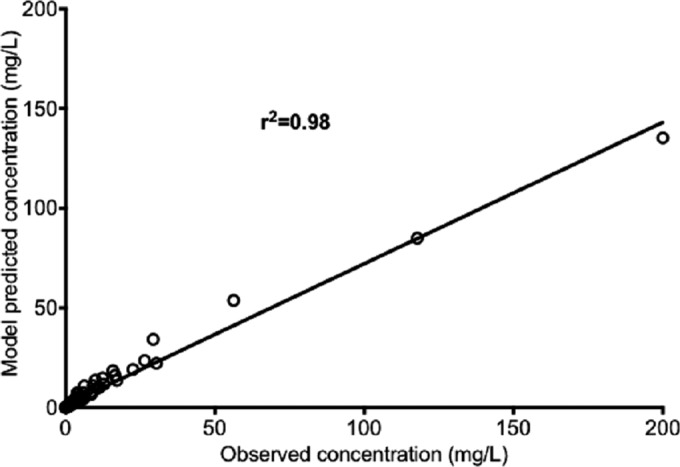
Amikacin concentrations as achieved in the HFS-TB. The two-compartment pharmacokinetic model predicted versus observed amikacin concentrations demonstrate a high *r*^2^ value, which means that the model described the concentration adequately.

Next we performed amikacin exposure effect studies in the HFS-TB using LPG M. tuberculosis at pH 6.8. The amikacin concentrations achieved in the HFS-TB were best described by a two-compartment pharmacokinetic model. The observed versus predicted concentrations in that model are shown in Fig. 3. Thus, we were able to recapitulate pharmacokinetics encountered in patients.

The nontreated M. tuberculosis controls in the HFS-TB grew at a rate of −0.11 (95% CI, 0.01 to 0.22) log_10_ CFU/ml/day (*P* < 0.04). The M. tuberculosis kill slope for all the amikacin regimens over the 28 days of treatment was 0.23 (95% CI, −0.29 to −0.17) log_10_ CFU/ml/day (*P* < 0.0001). The slopes for the six every-other-day dosing regimens were similar to those of their daily dose counterparts with matching peaks. There were no isolates resistant to amikacin at 6× MIC in the inoculum we used for the HFS-TB or in any drug-treated system at any time throughout the study, including in nontreated controls, which grew to 8.61 log_10_ CFU/ml on day 28.

**FIG 4:**
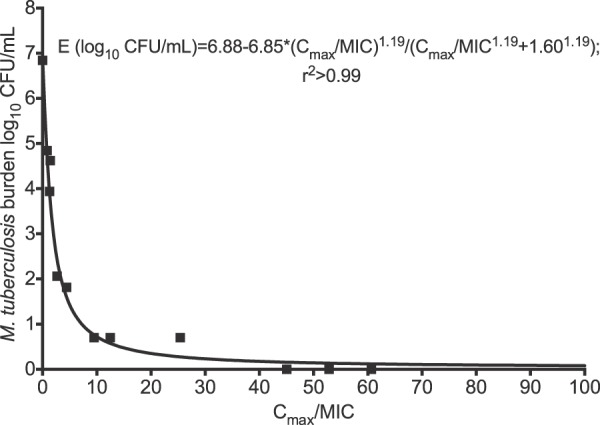
Amikacin peak concentration/MIC ratio versus microbial kill in the hollow-fiber system. The curve is for day14, with an *r*^2^ value of >0.99 for the relationship between *C*_max_/MIC ratio and bacterial burden, which means that this index is highly explanatory of the microbial effect.

Inhibitory sigmoid *E*_max_ modeling revealed that the lowest AIC scores of any sampling day for any of the PK/PD indices were on day 14, which also had the highest *r*^2^ values. Therefore, estimates on day 14 of the exposure effect relationships were used for further analyses. Figure 4 shows the regression for the *C*_max_/MIC ratio on that day. The AIC score was −18.81, which was the lowest for all PK/PD indices, making the *C*_max_/MIC ratio the variable linked to optimal M. tuberculosis kill. The exposure effect relationship depicted means that the EC_90_ is a *C*_max_/MIC ratio of 10.13 (95% CI, 7.76 to 12.48).

**FIG 5:**
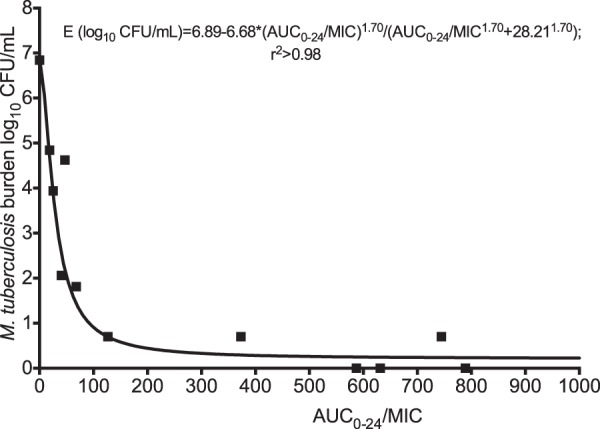
Amikacin AUC/MIC ratio versus microbial kill in the hollow-fiber system. The results are for day 14 bacterial burden versus AUC_0–24_/MIC ratio, with an *r*^2^ value of 0.98, so that the AUC/MIC ratio was also highly explanatory of effect, though less than the *C*_max_/MIC ratio.

The regressions for the AUC_0–24_/MIC ratio are shown in Fig. 5. The AIC score was −9.448. Based on this score, the relative likelihood that the *C*_max_/MIC ratio was a better choice than the AUC/MIC ratio was 106 times. The relationship shown in Fig. 5 calculates to an EC_90_ that is an AUC_0–24_/MIC ratio of 102.74 (95% CI, 77.72 to 127.80).

**FIG 6:**
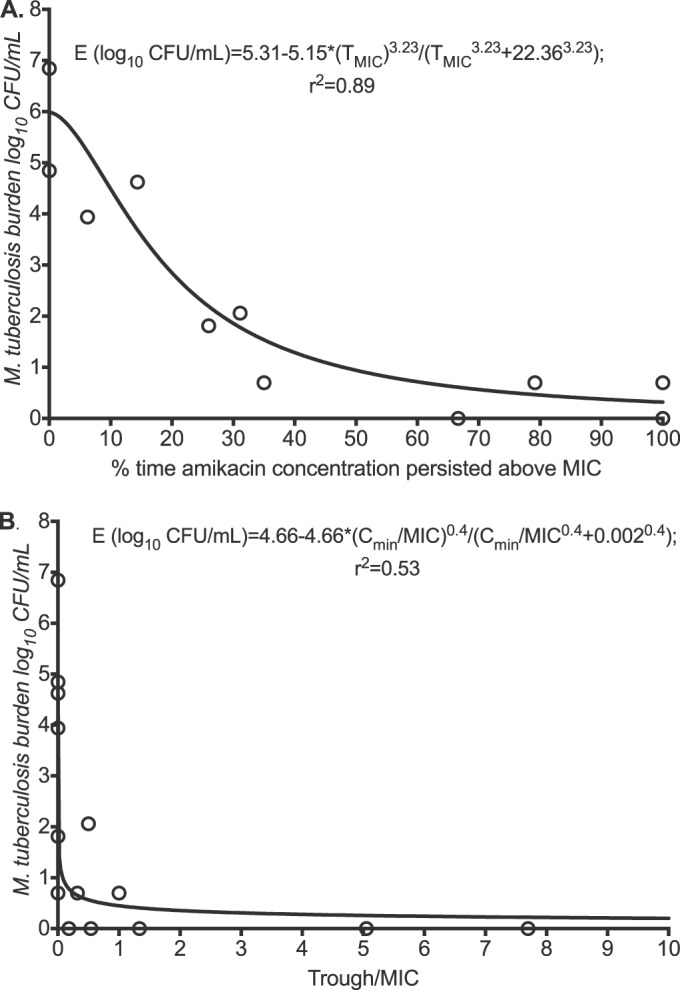
Amikacin time-driven indices versus microbial kill in the hollow-fiber system. The day 14 amikacin %*T*_MIC_ versus bacterial burden had a decent *r*^2^ value of 0.89 (A), but the *C*_min_/MIC ratio had a poor *r*^2^ value (B). Thus, the trough concentration explains very little of amikacin's efficacy.

The regression for %*T*_MIC_ is shown in Fig. 6A. The AIC score was 8.55. The relative likelihood that the *C*_max_/MIC ratio was a better choice than %*T*_MIC_ was 8.2 × 10^3^ times. Since the relative likelihood of %*T*_MIC_ being the PK/PD-linked parameter compared to the *C*_max_/MIC ratio was this low, we did not calculate an EC_90_ for %*T*_MIC_. Similarly, Fig. 6B shows the regression for the trough (*C*_min_)/MIC ratio as a PK/PD index. The AIC was 28.34.The relative likelihood that the *C*_max_/MIC ratio was a better choice than the *C*_min_/MIC ratio was 2.0 × 10^4^-fold, and thus we did not calculate an EC_90_ using this parameter.

## DISCUSSION

First, we report that contrary to standard belief for aminoglycosides, amikacin is likely to have sterilizing effect against SDB (24, 25). An acidic environment is known to impair the activities of many antibiotics; specifically, a 70-fold increase in the MIC has been observed for the aminoglycosides for a change in pH from 7.4 to 5.0 (26–28). In the historic study used for provenance of anti-TB chemotherapy in 1948, pulmonary TB patients were randomized to streptomycin monotherapy “2g per day, given in four injections at six-hourly intervals” (29). At 3 months, 8/54 of the streptomycin-treated patients had negative cultures, compared to 1/50 nontreated controls (*P* = 0.03), suggesting that in fact the drug had both bactericidal and sterilizing effects (29). In the seminal clinical study by Jindani et al., 1 g streptomycin once a day had an early bactericidal activity (EBA) of 0.12 log_10_ CFU/ml/day and a sterilizing effect rate of 0.18 log_10_ CFU/ml/day (30). On the other hand, more recently 10 mg/kg amikacin was reported to have no EBA at all (24). In our own longer-term studies with amikacin treatment in 437 MDR-TB patients in Botswana, 70% of patients had a favorable outcome but simultaneously had high rates of hearing loss; both rates were amikacin dose dependent (31). In the accompanying article, we show in a different cohort of MDR-TB patients from Botswana that rates of sputum conversion (which depends on both bactericidal effect and sterilizing effect) were amikacin concentration dependent (32). Thus, amikacin has both sterilizing and bactericidal effects.

Second, we identified that the PK/PD index linked to amikacin efficacy in the treatment of TB was the *C*_max_/MIC ratio. The AUC_0–24_/MIC ratio was also linked to efficacy, though the relative likelihood was 106 times less than for the *C*_max_/MIC ratio. Thus, similar to its activity against other bacteria and similar to the PK/PD of aminoglycosides as a class, microbial kill was concentration dependent. Studies by Mpagama et al. in Tanzanian MDR-TB patients, using the 2-hour concentration in their TB drug assay (TDA), showed that the amikacin 2-hour concentration/MIC ratio was linked to sputum conversion, consistent with a concentration-driven effect (33). In patients from Botswana, the highest-ranked predictor of sputum conversion in MDR-TB patients was serum *C*_max_, followed by AUC_0–24_ (32). On the other hand, we found that indices of time such as %*T*_MIC_ were less explanatory, with the *C*_min_/MIC ratio being even less useful. This means that the time-dependent indices such as trough play a minimal role in either efficacy (as shown here) or toxicity (as shown previously [16]) and are unimportant in monitoring therapy success or avoidance of toxicity in MDR-TB treatment. This also means that frequent daily parenteral dosing that includes weekends is not necessary; indeed, our every-other-day dosing scheme achieved kill slopes similar to those for daily dosing as long as the *C*_max_s were matched, which means that more intermittent injections would work as well as daily injections.

Third, we report the amikacin exposure associated with optimal efficacy. The optimal amikacin exposure in HFS-TB against M. tuberculosis was identified as a *C*_max_/MIC ratio of 10.13 ± 1.02. We have shown that the amikacin *C*_max_ associated with sputum conversion in MDR-TB patients in Botswana was 67 mg/liter in serum (32). Given the MIC distribution noted, this translates to a serum *C*_max_/MIC ratio of 67 to 89. Assuming an amikacin serum-to bronchial secretion ratio of 0.135 as well as negligible protein binding, this translates to a *C*_max_/MIC ratio of 9 to 12 in the lung (32), which is in the same range as our current findings in the HFS-TB. However, measures of aminoglycoside penetrations ratios are known to be variable, likely due to system hysteresis (34). Santre et al., for example, have identified a higher serum-to-bronchial secretion amikacin peak of 0.3, which would make the calculated optimal *C*_max_/MIC ratios about 2-fold higher; this would be counterbalanced, however, by the finding that >30% of patients had M. tuberculosis MICs of 2 mg/liter and higher (32, 35). Even with these variability factors, however, the range of *C*_max_/MIC ratio appears to be within range of that associated with optimal efficacy in patients. This value is also very close to the optimal *C*_max_/MIC ratio of 8 to 10 mg/liter identified for Gram-negative bacteria with amikacin and other aminoglycosides (36, 37).

This *C*_max_/MIC exposure target value can be used for individualizing therapy in TB programs. We propose that during the first week, the amikacin *C*_max_ be measured and the dose adjusted to the serum *C*_max_ of >67 mg/liter identified in patients, and then that as soon as amikacin MICs are available the dose be further adjusted to a serum *C*_max_/MIC ratio of 70 to 90. With this program we would advocate for identification of amikacin MICs for each MDR-TB patient's isolate using methods such as Sensititre MycoTB so that proper dose adjustment can be made for that patient, which would be true individualization of therapy. The main drawback of aminoglycoside treatment is nephrotoxicity and ototoxicity, which fortunately are associated not with either peak or trough concentration but with a cumulative serum amikacin AUC of 87,232 mg · h/liter (16). The dose, dosing frequency, and duration of therapy would be calculated to avoid achieving that cumulative AUC in each patient. The true starting doses for TB programs, the optimal times for pharmacokinetic samples, and the best dosing schedule for programs will, however, need to be specifically designed based on our current findings, and those on toxicity, using a Monte Carlo simulation exercise. That exercise is ongoing.

There are some limitations to our study. The first limitation is use of only one laboratory strain of M. tuberculosis in all our experiments, while the MICs for the clinical isolates vary. However, in the past, use of this one strain has been predictive of outcomes in patients. Indeed, in the accompanying article we show similar findings in MDR-TB patients (32). A second limitation is that there was no emergence of amikacin resistance in any of the regimens tested in the HFS-TB during the 28-day study. A likely reason is that our starting inoculum M. tuberculosis burden was below the inverse of the mutation frequency. Nevertheless, even then, the nontreated controls grew to a bacterial burden above this by day 28, still with no amikacin-resistant isolates. Another problem could be that in the assay for identification of the resistant subpopulation, the concentration of the drug was 6 times the MIC, which may be too steep. On the other hand, at least in regard to time to emergence of aminoglycoside resistance in the streptomycin monotherapy trial in 1948, resistance emerged after more than 1 month of therapy (29). Thus, this may simply be an artifact of the duration of our experiment. Finally, given that amikacin is given as part of combination therapy, the necessity to achieve the *C*_max_/MIC ratio of 10 for optimal kill will depend of the efficacy of companion drugs. If accompanying drugs have good bactericidal and sterilizing effects, therapy may still succeed even without achievement of the optimal amikacin *C*_max_/MIC ratio.

In summary, we identified potential sterilizing activity of amikacin, the *C*_max_/MIC ratio as the PK/PD index associated with amikacin efficacy, and a *C*_max_/MIC ratio of 10.13 in the HFS-TB as the optimal exposure target for effective individualized therapy against drug-resistant M. tuberculosis.
